# Impact of Antimicrobial Lipopeptides from *Bacillus* sp. on Suppression of Fusarium Yellows of Tatsoi

**DOI:** 10.1264/jsme2.ME15062

**Published:** 2015-06-27

**Authors:** Kenji Yokota, Hiroshige Hayakawa

**Affiliations:** 1 Department of Applied Biology and Chemistry, Tokyo University of Agriculture, 1–1–1 Sakuragaoka, Setagaya, Tokyo 156–8502, Japan

**Keywords:** *Bacillus*, Iturin A, Surfactin, *Fusarium*, Antimicrobial lipopeptide

## Abstract

Iturin A and surfactin are antimicrobial lipopeptides produced by antagonistic *Bacillus* spp. We herein demonstrated that both lipopeptides amended the soil-mediated suppression of the soil-borne disease, Fusarium yellows of tatsoi (*Brassica rapa* var. *rosularis*). Significant disease suppression was conferred by the amendments of purified iturin A or surfactin to soil. However, an excess amount of iturin A or surfactin to soil resulted in the loss of disease suppression activity.

*Bacillus* spp. are frequently isolated from the rhizosphere, and some beneficial strains have been identified as biocontrol agents for several plant diseases (2, 16, 18). Most *Bacillus* strains that confer disease suppression produce one or more lipopeptides with broad spectrum antimicrobial activity (1, 13). Iturin A and surfactin are cyclic lipopeptides produced by *Bacillus* spp. Iturin A is a heptapeptide (L-Asn → D-Tyr → D-Asn → L-Gln → L-Pro → D-Asn → L-Ser) linked to a β-amino fatty acid and consists of homologues A2 to A8, which differ by length and the structural isomer of the fatty acid (5). Surfactin consists of a heptapeptide (L-Glu → L-Leu → D-Leu → L-Val → L-Asp → D-Leu → L-Leu) linked to a β-hydroxy fatty acid, with homologues that also differ in length and the structural isomer of the β-hydroxy fatty acid (12). Both lipopeptides were recently reported to act as elicitors for the induced disease resistance of host plants as well as their antimicrobial activities (7, 9, 11).

The production of lipopeptides is considered to play a key role in the suppression of plant diseases by *Bacillus* strains. Romero *et al.*(14) reported that the production of iturin A or fengycin was critical for the suppression of melon powdery mildew caused by *Podosphaera fusca* because the biocontrol activities of the lipopeptide-deficient mutants were reduced. On the other hand, some *Bacillus* strains elicit the induced disease resistance of host plants through the production of some volatile compounds, *e.g.* 2,3-butanediol, which are different from lipopeptides (15). Since the mechanisms underlying disease suppression by *Bacillus* spp. are diverse, the contribution of lipopeptides to disease suppression by antagonistic *Bacillus* strains has not yet been elucidated in detail. Therefore, we herein investigated the effects of the purified lipopeptides, Iturin A and surfactin on the suppression of a soil-borne disease caused by *Fusarium oxysporum* f. sp. *rapae* on tatsoi, *Brassica rapa* var. *rosularis* (3).

Information regarding biological control including *Bacillus* spp. against Fusarium yellows and other diseases on tatsoi as the host plant is limited. Therefore, we evaluated disease suppression by lipopeptides. *F. oxysporum* f. sp. *rapae* (8), the causal agent of Fusarium yellows of tatsoi, was isolated by Dr. K. Ichikawa of Shizuoka Agricultural Experiment Station in Japan from the infected tissue of tatsoi. The seeds of *B. rapa* var. *rosularis* cv. Ryokusai No.2 were purchased from Sakata Seeds (Kanagawa, Japan).

Iturin A was purified from bacterial cultures of *B. subtilis* ATCC 21556 (6) as follows. One liter of a bacterial supernatant was extracted with 500 mL of ethyl acetate-1-butanol (7:3). The organic layer was collected and dried with a rotary evaporator. The residue was dissolved in methanol and applied to a silica gel column (Purif-Pack SI-30 size: 20, Shoko Scientific, Kanagawa Japan). Ethyl acetate-methanol (100:0 to 50:50 within 30 min by a linear gradient) was used as an eluent for silica gel column chromatography. The iturin A fraction was collected and dried with a rotary evaporator and dissolved in methanol for further purification by ODS column chromatography. ODS column chromatography was performed using ODS resin (Wakogel 50C18, Wako Pure Chemical Industries, Osaka, Japan) with a commercial purification kit (Shoko Scientific). Iturin A was eluted with methanol-DDW 50:50 for 30 min, followed by a linear gradient of 50:50 to 75:25 for 60 min and finally with 75:25 for 30 min. The purified iturin A after the ODS column was 98% pure and the molar ratio of 7 homologues of A2 to A8 was 0.9, 7.5, 35.8, 28.5, 1.4, 18.6, and 7.3, respectively, by a HPLC analysis (205 nm) (Fig. S1). Surfactin-Na derived from *B. subtilis* was purchased from Wako Pure Chemical Industries.

Microconidial suspensions were obtained from a liquid culture of the pathogen in Potato dextrose broth (Difco Laboratories Inc., Detroit, MI, USA) amended with 1.5 mM MgSO_4_·7H_2_O (17) by filtration through Kimwipes and collected by centrifugation at 8,000×*g* for 5 min at 4°C. The cell precipitate was suspended in sterilized water and counted using a Fucks-Rosenthal chamber.

Host plants were inoculated with the pathogen using a root-dip method (4). Tatsoi seedlings were germinated in an autoclaved potting mix (N:P_2_O_5_:K_2_O (mg L^−1^) = 320:210:300, 0.19 g cm^−3^, Takii seed, Kyoto, Japan) for 10 d. They were then removed and the roots washed under running tap water. The roots of seedlings were then dipped into the microconidial suspension for 30 s; control plants were dipped in sterilized water alone. The inoculated seedlings were transplanted into an autoclaved potting mix amended with lipopeptides. To achieve the amendment of lipopeptides in soil, iturin A or surfactin was dissolved in sterilized water and mixed into the soil. The plants were propagated in a growth chamber (27°C, light period 12 h, 100 μmol photons m^−2^ s^−1^). Disease severity was rated using a 0 to 3 rating scale (0, no disease; 1, wilt; 2, yellow; 3, dead). Thirty seedlings were used for each treatment. All experiments were repeated at least twice with similar results; the figures represent data from one such experiment.

The antifungal activity of iturin A or surfactin was evaluated in liquid cultures and soil. In liquid culture assays, microconidial suspensions obtained as described above were inoculated (OD_600_=0.05) into Czapeck Dox media with purified iturin A or surfactin at final concentrations ranging from 0 to 50 mg L^−1^. Two hundred microliters of these suspensions was then incubated in a 96-well microtiter plate at 25°C for 2 d. Fungal growth was evaluated by measuring OD_600_ with a SH-1000 microplate reader (Corona Electric, Ibaraki Japan). In soil assays, microconidia were inoculated into an autoclaved potting mix at a concentration of 1×10^4^ cells g dried soil^−1^. Lipopeptides were amended by mixing into soil at concentrations of 0.5 or 12.5 mg L soil^−1^ and incubated for 5 d at 27°C. The population size of *Fusarium* in soil samples was then determined by dilution plating on Komada’s *Fusarium*-selective medium (10) at 25°C by cultivation for 5 d.

Disease symptoms were observed within approximately 8 d post inoculation (dpi) in plants grown in control soils with the pathogen, but no amendment with lipopeptides (Table 1). Disease symptoms were delayed until 9 to 15 dpi and significant disease suppression was observed only at iturin A amendments of 0.47 mg L soil^−1^ in plants exposed to the 10^5^ conidia mL^−1^ pathogen inoculation (Table 1A). Disease suppression was also observed in plants exposed to the 0.47 mg L soil^−1^ and 10^6^ conidia mL^−1^ pathogen inoculation (Table S1A). Disease suppression was not observed when more than 0.94 mg L soil^−1^ of iturin A was added to soil when plants were exposed to either 10^5^ or 10^6^ conidia mL^−1^ of the pathogen (Table 1A and S1A). The dry weight of the above-ground parts of tatsoi grown in soil containing 3.75 mg L soil^−1^ iturin A, but without the pathogen inoculation was not significantly different from that of the healthy control (data not shown). The amendment of surfactin to soil also suppressed Fusarium yellows of tatsoi only when added to soil harboring 10^5^ conidia mL^−1^ pathogen at a concentration of 0.23 mg L soil^−1^; the addition of more than 0.47 mg L soil^−1^ did not yield disease suppression (Tables 1B and S1B).

In order to determine the antifungal activities of iturin A and surfactin, we evaluated their toxicities toward the fungal pathogen in liquid cultures and soil. In the liquid culture, growth inhibition by iturin A was observed at concentrations as low as 6.25 mg L^−1^ against the pathogen, and complete inhibition was noted at concentrations of 12.5 mg L^−1^ or higher. No inhibition of the pathogen was observed at any concentration of added surfactin, even as high as 50 mg L^−1^ (Fig. S2). The population size of the pathogen increased in soil from 1×10^4^ cells g dried soil^−1^ to 4×10^5^ CFU g dried soil^−1^ during the 5-d cultivation period (Fig. 1). The addition of Dazomet (Basmamide, Agro-Kanesho, Tokyo, Japan) at a concentration of 125 mg L soil^−1^ reduced the population sizes of the pathogen to below the limit of detection (<10^3^ CFU g^−1^) (Fig. 1). Although the treatments with iturin A or surfactin caused significantly lower population sizes than those with the pathogen control, the population sizes of the pathogen were increased during the cultivation up to the 12.5 mg L soil^−1^ treatment with iturin A or surfactin (Fig. 1).

Lipopeptides derived from *Bacillus* spp. have been suggested to play a key role in disease suppression against several kinds of plant diseases. In the present study, we demonstrated direct disease suppression by the purified lipopeptides iturin A and surfactin against a soil-borne disease caused by *Fusarium oxysporum* on tatsoi. Iturin A and surfactin both suppressed the *Fusarium* yellows of Taasai by soil amendments at relatively low concentrations (0.47 mg L soil^−1^ and 0.23 mg L soil^−1^, respectively) (Table 1). Higher concentrations did not confer disease suppression (Table 1).

Furthermore, although an inoculation with higher numbers of pathogen conidia caused more severe disease symptoms, the concentrations of lipopeptides that conferred significant disease suppression were independent of pathogen abundance. While the mechanism underlying disease suppression by iturin A is considered to be dependent on its antifungal activity (14), namely, disease severity should decrease with increasing amounts of iturin A in soil, our results are not consistent with such a mechanism because disease suppression was observed at intermediate concentrations of either iturin A or surfactin in the present study.

Our results suggest that the antifungal activity of iturin A may not contribute directly to its disease-suppressing activity. The concentration of iturin A needed to suppress fungal growth in a culture was markedly higher than that leading to disease control in soil. For example, the antifungal activity of iturin A against the pathogen was only observed at 6.25 mg L^−1^ (Fig. S2), whereas disease suppression was noted at 0.47 mg L^−1^ of soil (Table 1). In addition, we observed no antifungal activity of surfactin in either the liquid culture or soil, whereas it inhibited disease when added to soil (Table 1).

As discussed above, both lipopeptides have been shown to elicit induced disease resistance in host plants (7, 9, 11). The mechanism underlying disease suppression by the iturin A or surfactin treatment against Fusarium yellows of tatsoi in this study is consistent with the mechanism of induced disease resistance in the host plants. Furthermore, it is important to note that excess amounts of the lipopeptide treatments led to reduced disease suppression.

## Supplementary Information



## Figures and Tables

**Fig. 1:**
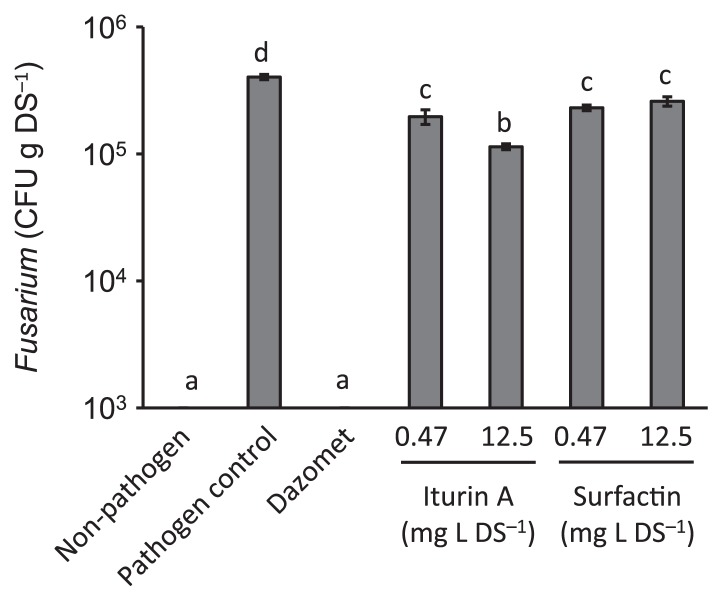
Antifungal activities of iturin A and surfactin for the pathogen of Fusarium yellows of tatsoi in soil. Error bars represent SE with 3 replications. Means followed by the same letter were not significantly different by Tukey’s test (α=0.05, *n*=3).

**Table 1:** Disease suppression by purified lipopeptide amendments against Fusarium yellows of tatsoi (10^5^ conidia mL^−1^ pathogen inoculation)

(A) Iturin A treatments									

Treatments	dpi								

	7	8	9	10	11	12	13	14	15
Healthy control	0.0±0.0	0.0±0.0	0.0±0.0**	0.0±0.0**	0.0±0.0**	0.0±0.0**	0.0±0.0**	0.0±0.0**	0.0±0.0**
Disease control	0.0±0.0	0.1±0.06	0.5±0.1	0.6±0.2	0.9±0.2	1.4±0.2	1.4±0.2	1.7±0.3	1.9±0.3
0.12 mg L^−1^ iturin A	0.0±0.0	0.03±0.03	0.2±0.1*	0.2±0.1*	0.6±0.2	1.1±0.2	1.3±0.2	1.7±0.3	1.8±0.3
0.23 mg L^−1^ iturin A	0.0±0.0	0.1±0.06	0.4±0.1	0.6±0.1	1.0±0.2	1.4±0.2	1.7±0.2	1.8±0.2	2.0±0.2
0.47 mg L^−1^ iturin A	0.0±0.0	0.0±0.0	0.07±0.05**	0.07±0.05**	0.2±0.1**	0.4±0.1**	0.6±0.2*	0.8±0.2*	0.9±0.2**
0.94 mg L^−1^ iturin A	0.0±0.0	0.07±0.05	0.3±0.1	0.4±0.1	0.8±0.2	1.2±0.2	1.6±0.2	1.9±0.2	2.0±0.2
1.88 mg L^−1^ iturin A	0.0±0.0	0.03±0.03	0.2±0.07	0.2±0.1	0.8±0.2	1.1±0.2	1.5±0.2	2.0±0.2	2.0±0.2
3.75 mg L^−1^ iturin A	0.0±0.0	0.1±0.06	0.4±0.1	0.5±0.1	1.1±0.2	1.4±0.2	1.7±0.2	2.0±0.2	2.0±0.2

(B) Surfactin treatments									

Treatments	dpi								

	7	8	9	10	11	12	13	14	15

Healthy control	0.0±0.0	0.0±0.0	0.0±0.0	0.0±0.0**	0.0±0.0**	0.0±0.0**	0.0±0.0**	0.0±0.0**	0.0±0.0**
Disease control	0.0±0.0	0.0±0.0	0.03±0.03	0.4±0.1	1.0±0.2	1.6±0.2	2.1±0.2	2.3±0.2	2.5±0.2
0.12 mg L^−1^ surfactin	0.0±0.0	0.0±0.0	0.07±0.05	0.3±0.08	0.6±0.2	1.1±0.2	1.5±0.2	1.8±0.3	1.9±0.3
0.23 mg L^−1^ surfactin	0.0±0.0	0.0±0.0	0.1±0.05	0.2±0.1	0.4±0.1*	0.6±0.2**	1.0±0.2**	1.2±0.2**	1.5±0.3**
0.47 mg L^−1^ surfactin	0.0±0.0	0.0±0.0	0.1±0.06	0.5±0.1	1.0±0.2	1.4±0.3	1.7±0.3	1.8±0.3	2.0±0.3
0.94 mg L^−1^ surfactin	0.0±0.0	0.0±0.0	0.3±0.08	0.6±0.1	1.0±0.2	1.4±0.2	1.8±0.3	2.0±0.3	2.2±0.2
1.88 mg L^−1^ surfactin	0.0±0.0	0.0±0.0	0.2±0.08*	0.5±0.1	0.7±0.2	1.2±0.2	1.5±0.2	1.9±0.2	2.1±0.2
3.75 mg L^−1^ surfactin	0.0±0.0	0.0±0.0	0.2±0.08*	0.4±0.1	1.1±0.2	1.4±0.2	2.2±0.2	2.2±0.2	2.3±0.2

Mean ± SE of disease severity are represented.

* significantly different at *P*<0.05 with Disease control by the Wilcoxon U-test;

** significantly different at *P*<0.01 with Disease control by the Wilcoxon U-test
