# Supplementary material for: Impact of Antimicrobial Lipopeptides from Bacillus sp. on Suppression of Fusarium Yellows of Tatsoi
Source: Microbes Environ. 2015 Jun 27;30(3):281–3. doi: 10.1264/jsme2.ME15062 (PMC4567569; doi:10.1264/jsme2.ME15062)
Supplement: Supplementary file 1 [file 30_281_s1.pdf]

Table S1 Disease suppression by purified lipopeptide amendment against Fusarium yellows of tatsoi (10^6 conidia mL^{-1} pathogen inoculation)

(A) Iturin A treatments

Treatments	dpi								
	7	8	9	10	11	12	13	14	15
Healthy control	0.0 ± 0.0	0.0 ± 0.0 **	0.0 ± 0.0 **	0.0 ± 0.0 **	0.0 ± 0.0 **	0.0 ± 0.0 **	0.0 ± 0.0 **	0.0 ± 0.0 **	0.0 ± 0.0 **
Disease control	0.0 ± 0.0	0.5 ± 1.0	1.0 ± 0.1	1.6 ± 0.2	2.4 ± 0.2	2.6 ± 0.1	2.7 ± 0.1	2.7 ± 0.1	2.8 ± 0.1
0.12 mg L^{-1} iturin A	0.0 ± 0.0	0.1 ± 0.1 **	0.4 ± 0.1 *	0.8 ± 0.2 **	1.5 ± 0.2 **	2.2 ± 0.2 *	2.4 ± 0.2	2.7 ± 0.1	2.8 ± 0.1
0.23 mg L^{-1} iturin A	0.0 ± 0.0	0.3 ± 0.1	1.0 ± 0.2	1.2 ± 0.2	1.9 ± 0.2 *	2.2 ± 0.2	2.5 ± 0.2	2.7 ± 0.2	2.7 ± 0.2
0.47 mg L^{-1} iturin A	0.0 ± 0.0	0.1 ± 0.1 **	0.3 ± 0.1 **	0.6 ± 0.1 **	1.0 ± 0.2 **	1.7 ± 0.2 **	2.0 ± 0.2 *	2.3 ± 0.2	2.5 ± 0.2
0.94 mg L^{-1} iturin A	0.0 ± 0.0	0.2 ± 0.04	0.7 ± 0.1	1.1 ± 0.2	1.7 ± 0.2 **	2.2 ± 0.2	2.4 ± 0.2	2.7 ± 0.2	2.8 ± 0.1
1.88 mg L^{-1} iturin A	0.0 ± 0.0	0.1 ± 0.1 **	0.7 ± 0.1	0.9 ± 0.2 **	1.5 ± 0.2 **	2.1 ± 0.2 *	2.4 ± 0.2	2.6 ± 0.2	2.7 ± 0.1
3.75 mg L^{-1} iturin A	0.0 ± 0.0	0.3 ± 0.1	0.8 ± 0.2	1.1 ± 0.2 *	1.9 ± 0.2	2.1 ± 0.2	2.4 ± 0.2	2.5 ± 0.2	2.6 ± 0.2

Mean ± SE of disease serverity are represented. *, significantly different at $P < 0.05$ with Disease control by Wilcoxon U-test; **, significantly different at $P < 0.01$ with Disease control by Wilcoxon U-test

(B) Surfactin treatments

Treatments	dpi								
	7	8	9	10	11	12	13	14	15
Healthy control	0.0 ± 0.0	0.0 ± 0.0 **	0.0 ± 0.0 **	0.0 ± 0.0 **	0.0 ± 0.0 **	0.0 ± 0.0 **	0.0 ± 0.0 **	0.0 ± 0.0 **	0.0 ± 0.0 **
Disease control	0.0 ± 0.0	0.2 ± 0.08	0.7 ± 0.1	1.2 ± 0.1	1.8 ± 0.2	2.1 ± 0.2	2.5 ± 0.2	2.7 ± 0.1	2.8 ± 0.1
0.12 mg L^{-1} surfactin	0.0 ± 0.0	0.2 ± 0.08	0.7 ± 0.1	1.1 ± 0.1	1.8 ± 0.2	2.6 ± 0.1	2.9 ± 0.1 *	3.0 ± 0.03	3.0 ± 0.0
0.23 mg L^{-1} surfactin	0.0 ± 0.0	0.2 ± 0.08	0.4 ± 0.1	1.0 ± 0.2	1.4 ± 0.2	1.9 ± 0.2	2.4 ± 0.2	2.6 ± 0.2	2.7 ± 0.2
0.47 mg L^{-1} surfactin	0.0 ± 0.0	0.1 ± 0.06	0.4 ± 0.1	1.0 ± 0.2	1.6 ± 0.2	2.4 ± 0.2	2.8 ± 0.1	2.9 ± 0.1	2.9 ± 0.1
0.94 mg L^{-1} surfactin	0.0 ± 0.0	0.0 ± 0.0 **	0.4 ± 0.1	1.0 ± 0.2	1.8 ± 0.3	2.7 ± 0.1	2.8 ± 0.1 *	2.9 ± 0.1	2.9 ± 0.1
1.88 mg L^{-1} surfactin	0.0 ± 0.0	0.3 ± 0.08	0.8 ± 0.1	1.7 ± 0.1 **	2.3 ± 0.2	2.8 ± 0.1 *	3.0 ± 0.0 **	3.0 ± 0.0 *	3.0 ± 0.0
3.75 mg L^{-1} surfactin	0.0 ± 0.0	0.1 ± 0.06	0.6 ± 0.1	1.5 ± 0.2	2.1 ± 0.2	2.5 ± 0.2	2.8 ± 0.1	2.9 ± 0.1	2.9 ± 0.1

Mean ± SE of disease serverity are represented. *, significantly different at $P < 0.05$ with Disease control by Wilcoxon U-test; **, significantly different at $P < 0.01$ with Disease control by Wilcoxon U-test

Fig. S1. HPLC chromatogram of purified iturin A. Peaks 1 to 7 correspond to iturin A2 to A8, respectively.

Fig. S2. Antifungal activities of iturin A and surfactin for the pathogen of Fusarium yellows of tatsoi in a liquid culture.

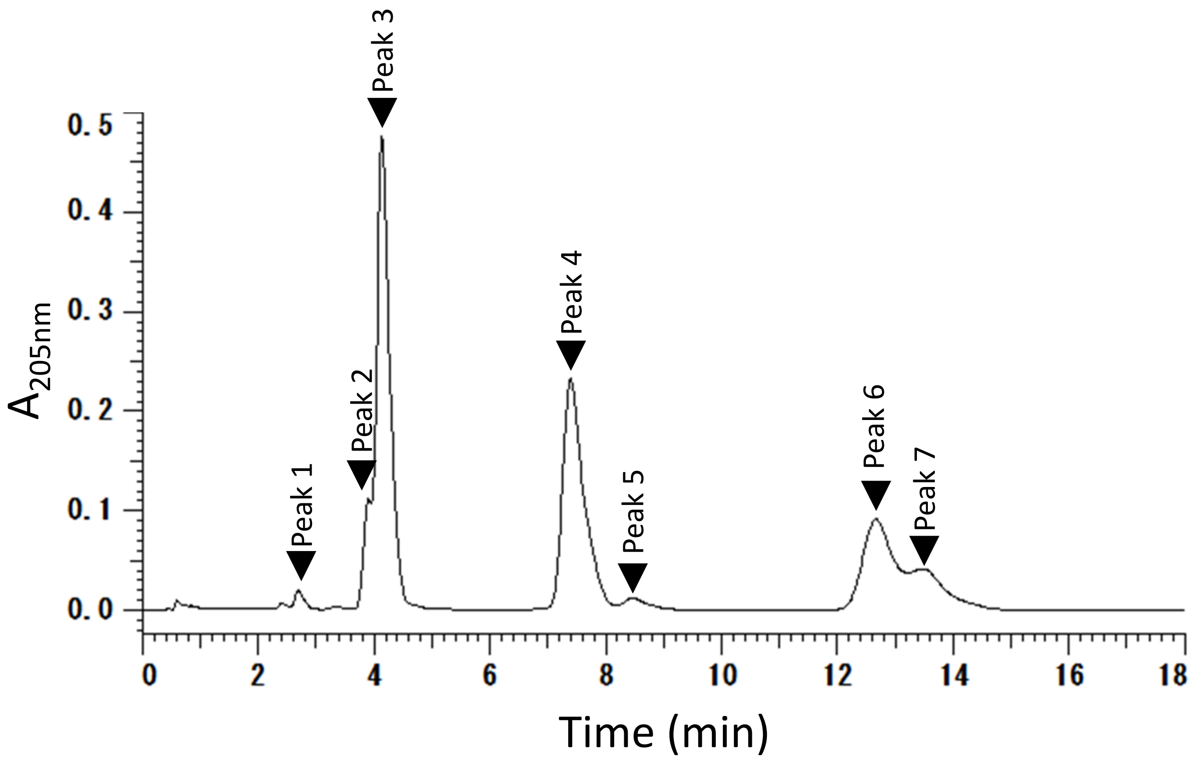

Fig. S1. Yokota

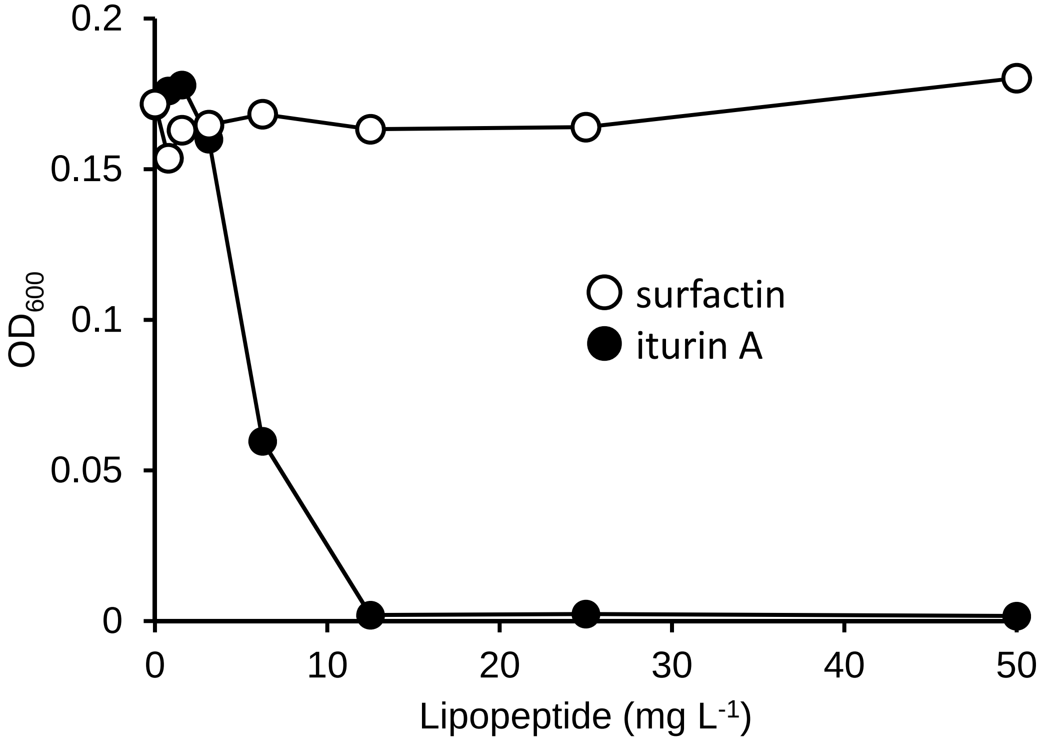

Fig. S2. Yokota
